# Hesperetin-7-*O*-glucoside/β-cyclodextrin Inclusion Complex Induces Acute Vasodilator Effect to Inhibit the Cold Sensation Response during Localized Cold-Stimulate Stress in Healthy Human Subjects: A Randomized, Double-Blind, Crossover, and Placebo-Controlled Study

**DOI:** 10.3390/nu15173702

**Published:** 2023-08-24

**Authors:** Mahendra P. Kapoor, Masamitsu Moriwaki, Aya Abe, So Morishima, Makoto Ozeki, Norio Sato

**Affiliations:** 1 Nutrition Division, Taiyo Kagaku Co., Ltd., 1-3 Takaramachi, Yokkaichi 510-0844, Mie, Japan; 2 Taiyo Kagaku Co., Ltd., 800 Yamada-Cho, Yokkaichi 510-1111, Mie, Japan

**Keywords:** hesperetin, inclusion complex, cold sensation, localized cold-stimulated stress, skin temperature, skin blood flow, vasodilator

## Abstract

Hesperetin, a citrus flavonoid, exerts vasodilation and is expected to improve endothelial function and alleviate cold sensation by activating nervous system thermal transduction pathways. In this randomized, double-blind, crossover, and placebo-controlled study, the purpose was to assess the effect of an orally administered highly bioavailable soluble inclusion complex of hesperetine-7-*O*-glucoside with β-cyclodextrin (HEPT7G/βCD; SunActive^®^ HES/HCD) on cold sensation response during localized cold-stimulated stress in healthy humans. A significant (*p* ≤ 0.05) dose-dependent increase in skin cutaneous blood flow following relatively small doses of HEPT7G/βCD inclusion complex ingestion was confirmed, which led to a relatively effective recovery of peripheral skin temperature. The time delay of an increase in blood flow during rewarming varied significantly between low- and high-dose HEPT7G/βCD inclusion complex consumption (e.g., 150 mg and 300 mg contain 19.5 mg and 39 mg of HEPT7G, respectively). In conclusion, the substantial alteration in peripheral skin blood flow observed during local cooling stress compared to placebo suggested that deconjugated hesperetin metabolites may have a distinct capacity for thermoregulatory control of human skin blood flow to maintain a constant body temperature during cold stress exposure via cutaneous vasodilation and vasoconstriction systems.

## 1. Introduction

Thermosensation is a sensory process. Harsh environmental conditions can rapidly lead to harmful hypothermia or hyperthermia [1]. Proper blood circulation is crucial for the body because it enables the transport of oxygen and nutrients to cells. Healthy blood flow helps regulate body temperature and maintain a certain pH level to sustain bodily functions. In turn, biological systems symbolize a peculiar type of control system, wherein the hands and feet act as powerful thermoregulatory regulators such as heat exchangers and thermal insulators and enable us to swiftly react to noxiously hot and cold environments, respectively [2]. Thus, thermoregulation is a fundamental capacity of the autonomic nervous system to respond to cold and heat stress conditions [3]. Prolonged local exposure of the extremities to a cold environment can result in cold stress-related injuries [4,5,6,7], possibly due to a sympathetically mediated vasoconstriction response with arteriovenous anastomoses. Decreasing local skin temperature causes temperature-dependent vasoconstriction, resulting in reduced blood flow to the peripheries in favor of a central pooling of blood in the torso and deep core of the extremities. Blood viscosity seems to increase 2% per degree centigrade drop in temperature [8]. In addition, the thermal responses during passive recovery and the ensuing cold stress have also been considered to evaluate the subjective cold sensation that is susceptible to cold-stimulated injuries [9,10,11,12], possibly represented by an abrupt increase in the skin temperature due to the cold-stimulated vasodilation response. Hence, normal arterial function requires a balance between vasoconstriction and vasodilation, which is important for regulating blood flow in cold-sensation conditions.

In general, the cold sensation is the feeling of coldness at temperatures that are normally not considered so cold by the majority of people. Such epidemiology (so-called *hie*) is common in Japan and is experienced by nearly 60% of the Japanese population, including mostly women (94.6%) in the age range of 15–65 years [13]. Generally, the cold sensation is believed to occur because of poor blood circulation due to an abnormality or bias in the balance between heat production or transport and the rate of heat dissipation. The cold sensation can also accompany several other uncomfortable symptoms, such as shoulder stiffness, numbness and tingling in the hands and feet, fatigue, muscle cramping and lumbago, varicose veins, hot flushes, and difficulty concentrating. In addition, poor blood circulation may also induce changes in skin color or digestive problems (e.g., constipation) [14].

Certain dietary foods (such as seasonal fruits, beets, nuts, garlic, red-hot peppers, and some sea foods) play a vital role in blood flow [15,16]. Phytonutrient flavonoids may increase blood flow sufficiently to warm the hands, feet, and body of those with subjective cold sensitivity and possessing a low or high risk of cold injury [17]. However, limited reports are available, and what is involved in stimulating such effects remains unanswered. A very small proportion of flavonoids ingested are absorbed in the body because their poor solubility in water greatly limits their bioavailability after oral administration [18,19,20]. Once ingested, following absorption in the epithelial cells of the small intestine where phase I metabolism occurs, the flavonoids form conjugated metabolites (phase II metabolites) in the large intestine by colonic microflora, which enter the blood circulation through the portal vein [21,22]. Parez et al. investigated the acute vasodilator effects of the flavonoid quercetin in healthy subjects [23]. The results were consistent with the effect being due to the deconjugation of the quercetin metabolites in correlation with beta-glucuronidase activity. Furthermore, the endothelium plays a major role in the regulation of blood flow. The release of nitric oxide (NO) by endothelial cells causes the dilation of an artery, which leads to increased blood flow [24,25].

Hesperidin and its aglycone have been studied for their potential health benefits, including their ability to improve blood flow in the body by reducing inflammation and oxidative stress in the body, which can improve the health of blood vessels. The flavonoids hesperidin and hesperetin increase endothelial function via increased NO production, synthesized by the enzyme endothelial nitric oxide synthetase (NOS) [26], a molecule that helps relax blood vessels and improve blood flow, and decreased monocyte adhesion in the endothelial cells that line the interior surface of blood vessels and play a crucial role in regulating blood flow [27,28,29,30]. Recent studies suggest that hesperetin may help alleviate cold sensations in the body [31,32]. Although hesperidin and hesperetin show promising potential as natural ways to improve blood flow, additional research is needed to fully understand their effects and mechanisms, as well as the potential benefits of hesperetin for cold sensations [33].

In this study, we performed a short-term cold provocation test among healthy individuals to confirm the efficacy of a highly soluble proprietary formulation of hesperetin-7-*O*-glucoside inclusion complex with β-cyclodextrin (HEPT7G/βCD), wherein a glucoside derivative of hesperetin was encapsulated in the hydrophobic cavity of β-cyclodextrin [34]. We examined the effect of oral ingestion of the highly bioavailable HEPT7G/βCD inclusion complex [35] on peripheral blood flow and skin surface temperature in healthy human subjects following localized cooling-induced stress in the randomized, double-blind, crossover, and placebo-controlled study. It is believed that the endothelial function of hesperetin glycosides may modulate its efficacy on the recovery of peripheral body temperature and promote blood flow to counteract cold sensation conditions in healthy human subjects. The study evaluated peripheral skin blood flow as the primary endpoint, while peripheral body temperature and questionnaire-based subjective symptoms were considered secondary endpoints. Additionally, any adverse events and side effect symptoms were examined as safety endpoints. We hypothesized that the oral HEPT7G/βCD inclusion complex might induce vasodilator effects in humans and might be related to the deconjugation of hesperetin-glucuronide metabolites. The HEPT7G/βCD inclusion complex could be a valuable vasodilator to manage human physiology, body temperature homeostasis, and thermoregulatory responses in extreme conditions because of the consequent changing global temperature.

## 2. Materials and Methods

### 2.1. Study Design, Study Participants, and Preliminary Section Criteria

In this randomized, double-blind, crossover, and placebo-controlled intervention study, 42 healthy Japanese adult volunteers (*n* = 42; 14 male (M) and 28 female (F)) aged between 20 and 65 years were enrolled and evaluated at LSI Sapporo Clinic, Hokkaido, Japan, by IMEC RD Co., Ltd., Tokyo, Japan. The study protocol was initially reviewed and approved by the ethical committee expert group of Taiyo Kagaku Co., Ltd., Japan, and subsequently approved by the Suda Clinic institutional review board of IMEC RD Co., Ltd., Tokyo, Japan (approval No. IQRD-05-022; date: 19 January 2022). Finally, the protocol was registered at the University Hospital Medical Information Network-Clinical Trial Registry (UMIN-CTR; Trial ID: *UMIN 000046806*; date: 4 February 2022) and followed without modification from the time of final approval and during the study. The study was performed following the revised guidelines for Medical Research Involving Human Subjects (Ministry of Education, Culture, Sports, Science and Technology, and the Ministry of Health, Labor, and Welfare, 2021) and using methodologies that fully complied with all ethical standards principles laid out in the Helsinki Declaration and its amendments [36].

The enrolled participants were well informed about the purpose of the study, including privacy protection, health hazards and risks, compensation, data management, etc., and the procedures and contents of the study were verbally explained. Informed consent was signed by all participants prior to the preliminary checkup and screening for inclusion and exclusion criteria. Enrolled subjects were asked to complete a preliminary health screening form, and to be eligible, the following criteria had to be met: (i) 20 years of age or older and computer literate; (ii) body mass index (BMI) < 30 kg/m^2^; (iii) no history of major illness, recent surgery, or long-term medical conditions; (iv) non-smokers; (v) not under any medication or taking health-promoting foods and supplements, diet, or exercise therapy; (vi) do not perform vigorous exercise regularly; (vii) reported to be regularly menstruating (female subjects only; participated in study 2 weeks after the menstrual period to avoid fluctuation of basal body temperature); and (viii) have not participated in other clinical studies in the month before the start of this study. Exclusion criteria were: (i) subjects with a high habitual consumption of caffeine (>100 mg/day) and alcohol (>20 g/day); (ii) unstable dietary regimen/history; (iii) pregnancy and breastfeeding; (iv) have used nutraceutical products or pharmaceuticals that influence blood circulation, and related to fatigue, stress relief, sensitivity to cold, blood pressure, warming effect, etc.; (v) subjects with current or past drug or food allergies; (vi) subjects who donated a maximum amount of blood (male: 400 mL or more; female: 200 mL or more) within 3 months before obtaining informed consent; (vii) subjects who work in shifts or who plan to travel abroad during the research period; and (vii) subjects declared ineligible by the medical practitioner or principal investigator of this study.

**Figure 1:**
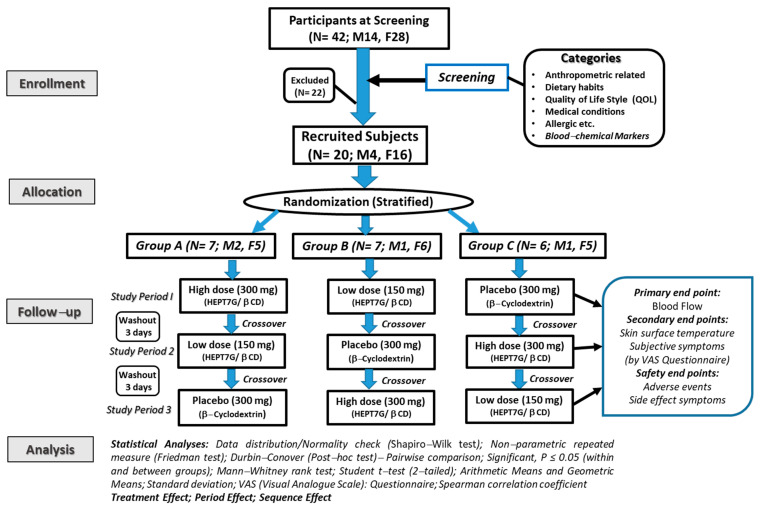
A schematic representation of the CONSORT flow diagram of the protocol and a detailed description of the study in healthy human subjects.

A questionnaire comprising dietary food intake, quality of life, medical conditions, bowel habits, general lifestyle, sleep conditions, health supplement uses, and an allergic statement was distributed to participants who expressed interest and enrolled in this study two weeks before the start of the screening process. Subjects were evaluated for cold sensation conditions using Terasawa’s diagnostic criteria, as reported elsewhere [37]. The outlines of the diagnostic are listed in the Supplementary Information (see Table S1). The study subjects were recruited based on their preclinical test results of peripheral blood flow and body surface temperature, along with their response to the cold sensation condition diagnostic criteria. After a sample size calculation that estimated the prerequisite of approximately 18–20 participants for this study, the required 20 subjects (*n* = 20) were recruited for this study. A set enlisting strategy as per protocols was followed, so the recruited study subjects could be conveniently handled at the designated clinical research facility. Finally, the recruited subjects received an explanatory document and a copy of the duly signed consent certification of their participation in this study. Further, the recruited subjects (*n* = 20) were randomly allocated to three separate groups using a block randomization design stratified by gender, the recovery time of peripheral blood flow, and body surface temperature after localized cooling stress. The allocation was performed by an independent researcher of IMEC RD Co., Ltd., Tokyo, Japan, who was practically not involved in the study planning, protocols, conduct, or analysis. As per protocol, the allocation was blinded and remained concealed from the study subjects, the general instructor, the data management and statistical analyst, and the principal investigator until the completion of the study. The key coding of the groups that received either high or low dosages of treatment or placebo was revealed to researchers only after all measurements were completed and reported to finalize the study outcomes. A schematic illustration of the experimental design of the study protocol is presented in Figure 1.

### 2.2. Study Test Materials, Supplementation, and Dosages

The test dietary materials were a proprietary hesperetin-7-*O*-glucoside inclusion complex with β-cyclodextrin [HEPT7G/βCD inclusion complex: SunActive^®^ HES/HCD; HEPT7G content: 14.2–14.5% (*w*/*w*); β-CD content: 57.5% (*w*/*w*); saccharides content: 24%; moisture 6%; manufacturer: Taiyo Kagaku Co., Ltd., Yokkaichi, Japan], and the placebo β-cyclodextrin (high purity β-CD; a product of Wacker Chemical Corporation, Chino, CA, USA) was purchased from Cyclo Chem Co., Ltd., Kobe, Japan. The formulation, structure evaluation, bioavailability, and details on clinical safety of the HEPT7G/βCD inclusion complex are already reported elsewhere [34,35,38].

The study was a three-period crossover trial, wherein subjects of each group consumed the placebo and both a high dose (300 mg) and a low dose (150 mg) of the HEPT7G/βCD inclusion complex during three consecutive periods in a crossover manner, allowing a washout period of at least three days. Based on the safety test results concerning the HEPT7G/βCD inclusion complex [38], the chosen dosages are considered safe for single administration.

Hard hydroxypropyl methylcellulose (HPMC) capsules were prepared to contain a placebo and both high and low doses of the HEPT7G/βCD inclusion complex and were identical in appearance, size (17.2 mm), color (light brown), and packaging. Calcium stearate (CAS 1592-23-0; MW 607.02 g/mol) at a concentration of 1.0% was used as a lubricant with anti-adherent properties. The specifications of the active raw ingredient and placebo were confirmed by an independent laboratory of a capsule manufacturer (Japan Tablet Co., Ltd., Kyoto, Japan). The placebo capsule contained only β-CD, whereas, the high-dose active ingredient capsule contained 150 mg of HEPT7G/βCD inclusion complex [i.e., 19.50 mg of HEPT7G equivalent], and the low-dose active ingredient capsule contained 75 mg of HEPT7G/βCD inclusion complex [i.e., 9.75 mg of HEPT7G equivalent]. The weight of the capsules was adjusted based on the density and amount of β-CD.

### 2.3. Study Protocol, Procedure, Instrumentation, and Measurements

The study was conducted between January and March 2022 to eliminate the seasonal heat effect on the study measurements. As per protocol, the participants arrived at the designated clinical study facility at noon (i.e., 12:00 h) after having abstained from lunch or any food and beverage for at least 2 h before the start of the experimental measurements. Participants were also asked to abstain from exercise, caffeine, and alcohol for at least one day. Each measurement was performed without lunch between 12:00 and 14:30 h to avoid any influence of diurnal changes in the body temperature.

Participants wore light, comfortable clothing for each measurement to eliminate the possibility of the clothing’s influence on body surface temperature and other measured parameters. The participants were quietly seated in the measurement chamber for 15 min to acclimate them to the constant ambient conditions within the measurement room. The mean temperature and relative humidity in the measurement chamber were 25 ± 1 °C and 30.0 ± 5%, respectively. After becoming accustomed to the experimental chamber conditions, the participants were given the two study capsules, each containing either a placebo or varied doses (i.e., 300 mg and 150 mg) of HEPT7G/βCD inclusion complex with 100 mL of distilled water for oral ingestion. The doses and timing of their intake were selected because they can produce a significant increase in plasma hesperetin metabolite levels within the physiological range. After 45 min from time of intake, the measurements were carried out with the study participants calmly resting in a spine position throughout each testing period. The order of the experiments was randomized at intervals of three days during the washout period. Whereas, for the initial screening, the same protocol was performed except that the participants were given only 100 mL of distilled water as pre-ingestion.

**Figure 2:**
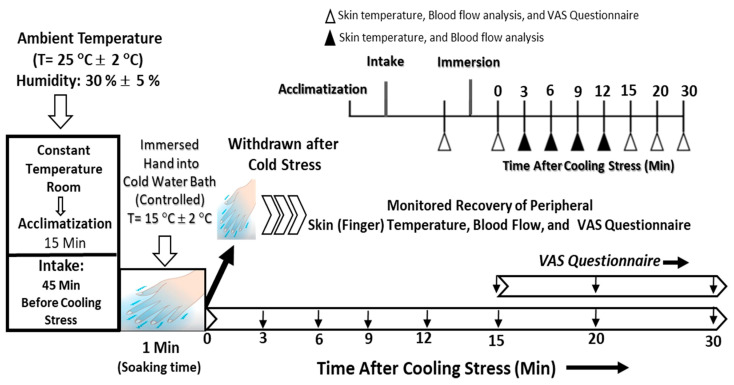
A conventional schematic of the study protocol procedure to assess the efficacy of the proprietary HEPT7G/βCD inclusion complex on the peripheral skin temperature and blood flow during cold-stimulate stress and subsequent recovery after cooling stress in healthy human subjects.

The peripheral skin temperature of the middle finger on the left hand was measured using a Type-K thermometer equipped with a sensor probe sealed with non-permeable tape. After that, the left hand was immersed up to the ulnar and radial styloid in a tank containing cold water precisely maintained at 15 ± 2 °C by means of a cooling system controller, and a pump continuously stirred the water. The cooling stress was maintained for one minute, and then the hand was removed from the cold water tank, and a spontaneous rewarming phase ensued with continued measurement of the peripheral skin temperature of the finger at regular intervals for the following 30 min upon completion of the cooling stress to assess the temperature recovery, i.e., rewarming (Figure 2). Blood flow was monitored using a laser-Doppler blood flow meter (RBF-101/P101; Pioneer Corporation, Tokyo, Japan; wavelength 850 nm; temperature range 10–40 °C; and 30–75% relative humidity) equipped with a high-sensitivity integrating sensor probe, housed in a 3 cm diameter heater element placed on the skin. The cutaneous skin blood flow in the annular finger of the left hand was measured simultaneously with the measurement of the peripheral skin temperature of the middle finger on the left hand throughout the entire protocol. During the test, the study subjects were instructed to keep their contralateral hand and body immobile while resting in a sitting position.

During the visit to the clinical trial facility, the height was measured using a calibrated portable stadiometer with an accuracy of 0.1 cm (Seca, Japan), and body mass (weight) was measured using a digital weighing scale with a sensitivity of 0.1 kg (DP-7200, Daiwa Seihei Co., Ltd., Tokyo, Japan) for each participant. The body mass index was estimated as body weight in kilograms divided by height in meters squared. The blood pressure and pulse rate were recorded using a sphygmomanometer (a digital blood pressure monitor) equipped with a double air bladder and an irregular pulse wave detection function (Omron HEM-1000; Omron Corporation, Kyoto, Japan). All measured variables were recorded by a data logger.

### 2.4. Subjective Symptom Assessments

The information concerning the subjective symptoms of study participants was collected using a visual analog questionnaire (VAS), where the leftmost and rightmost sides represented no symptoms and the severest symptoms, respectively, on the VAS horizontal line of 100 mm in length. Participants were required to respond to queries related to subjective sensations. Three major questions related to cold sensation symptoms, such as ratings on the cold sensation of the fingertips, hand, and whole body, were chosen to diagnose the classified subjective cold sensation among the study participants. Other questions were related to diagnosing cold sensations in the toes and feet.

### 2.5. Data Processing and Statistical Analyses

Statistical analyses were performed using JMP v.14 (the SAS software package). Data distributions were viewed by examining the normal quantile plots and histograms, and normality was assessed using the Shapiro–Wilk W test (a goodness-of-fit test). Study variables are expressed as mean ± SD unless otherwise indicated, whereas VAS data that were not normally distributed were presented as the geometric mean ± SD. Variables that were not normally distributed were subjected to non-parametric statistical analysis. We used a non-parametric repeated measure Friedman test to assess the differences in peripheral skin temperature and skin blood flow scores across three conditions: high dose, low dose, and placebo. The investigation hypothesis point was whether there would be a significant difference in peripheral skin temperature and skin blood flow scores between the three conditions. The Friedman test is a non-parametric alternative to repeated-measures ANOVA, which is recommended to be used to analyze related data when the dependent variable is measured on an ordinal scale. It is a non-parametric test, which entails that it does not assume that the data are normally distributed or have equal variances. The significance level was set a priori at *p* ≤ 0.05 and checked to ensure that the data met the assumptions of a non-normal distribution before conducting the repeated-measures Friedman analysis. Any outliers or missing data points were checked prior to analysis. The test statistic we used was the Friedman Chi-squared statistic, and we used the Durbin–Conover post-hoc test to compare the differences between pairs of conditions (pairwise comparison by Z-score) if a significant main effect was observed. Since post-hoc tests differ in their assumptions and the level of significance they use, we chose the Durbin–Conover test, which is the most appropriate post-hoc test for our study hypothesis. Since the effect of localized cooling in the hematologic system consists of a simultaneous increase in the subject’s hemoglobin and hematocrit levels, a stratified analysis of the variation in peripheral skin temperature of the finger was performed by creating a subgroup within the baseline hemoglobin concentration in the normal level range (i.e., >13.2 g/dL). The sequence effect and period effect were also examined, and the data is presented as the geometric mean ± SD. We conducted the repeated measure Friedman test analyses using Jamovi (2.2.5) statistical software. Since VAS scores were different between participants, the scores were transformed for easier comparison and normalized within each participant by dividing each participant’s original VAS value by the maximum VAS possible prior to statistical analysis. Additionally, a non-parametric Spearman correlation coefficient was used to evaluate the relationship between the selected study variables.

## 3. Results

### 3.1. Study Characteristics and Procedural Analyses

A total of 42 (*n* = 42; male 14, female 28) subjects were initially enrolled in the study, but after the screening, 22 (*n* = 22) subjects were excluded based on set inclusion and exclusion criteria. Finally, 20 (*n* = 20; M4, F16) subjects were recruited for this study. The average age was 47.6 ± 8.5 years, and the average body mass index (BMI) was 22.1 ± 2.4 kg/m^2^. The selected subjects (*n* = 20) were randomized into the three study groups using a block randomization design stratified by gender, the recovery time of peripheral blood flow, and body surface temperature after localized cooling stress. No significant differences in anthropometric parameters, i.e., age, height, body weight, and BMI, including other parameters, were noticed between the groups (*Group A*: *n* = 7, M2, F5, *Group B*: *n* = 7; M1, F6, and *Group C*: *n* = 6; M1, F5) at baseline. The recovery of the blood flow ratio and skin surface temperature ratio among groups were also compared at baseline and showed no difference between the groups, confirming the homogeneity among the groups. The primary baseline characteristics and blood test parameters at baseline of the study subjects are presented in Table 1. In this three-period crossover study, subjects in each group consumed the acute dose of placebo and both the high dose (300 mg) and low dose (150 mg) of the HEPT7G/βCD inclusion complex during three consecutive periods in the crossover model, allowing a washout period of at least three days. All selected subjects completed the study and fully complied with the study protocols. A complete set of the data collected according to the protocol procedures is included in the data analysis for the study outcomes. Additionally, based on the use of a two-sample comparison of means at the alpha (α) = 0.05 level of significance, the total sample size of each group provided >85% power to detect an appropriate effect size.

Normality, equal variance, assumptions about the distribution of the data, or linearity were checked to confirm the use of a non-parametric statistical method. The normality of the data was assessed using the Shapiro–Wilk test (appropriate for small sample sizes of 50 samples or less). The results indicated that the data was not normally distributed (W = 0.91; *p* < 0.05). Therefore, a non-parametric statistical repeated-measures Friedman rank test method, which is more robust to outliers and extreme values, was applied for the subsequent analyses because the data does not meet the assumptions of parametric methods. The distribution of the data as a histogram revealed that the data is skewed to the right, with a long tail on the positive side of the distribution. Such a deviation from normality further suggests that a non-parametric test may be more appropriate for analyzing the data.

### 3.2. Skin Peripheral Blood Flow and Temperature Profiles upon Cold-Stimulated Stress

**Figure 3:**
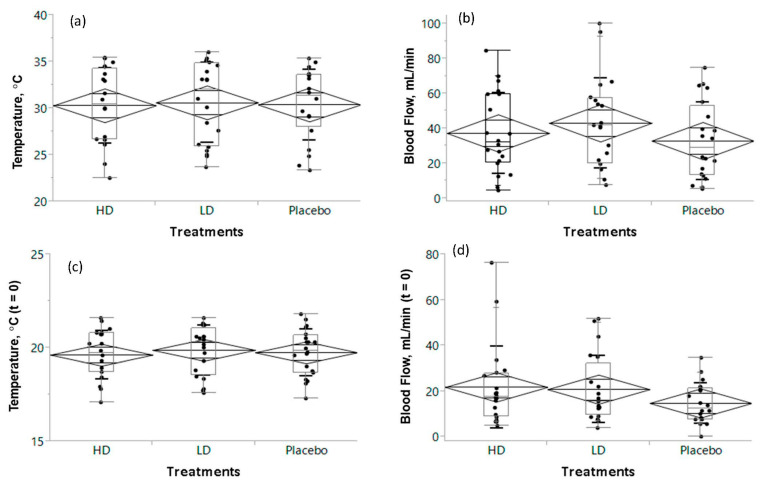
The skin peripheral temperature and blood flow profiles of the study subjects just before the cold-stimulate stress ((**a**,**b**); temperature and blood flow, respectively), and just after the cold-stimulate stress, i.e., t = 0 ((**c**,**d**); temperature and blood flow, respectively). The box plot of median and mean values (diamond shape) of profiles is presented for placebo, high dose (HD), and low dose (LD) HEPT7G/βCD inclusion complex intake groups; significant: *p* ≤ 0.5.

During the screening, the baseline autonomic and subjective responses of the participants for finger skin blood flow and temperature did not differ in the cold-stimulate stress tests. The skin peripheral blood flow and temperature profiles of the study subjects just before the cold-stimulate stress are presented in Figure 3.

The box plot of median and mean values (diamond shape) of profiles revealed that the finger skin temperature did not differ (30.3 ± 4.0, 30.6 ± 4.3, and 30.4 ± 3.8 °C after ingestion of either a high dose or a low dose of the HEPT7G/βCD inclusion complex, or placebo, respectively) just before immersion of the hand into a cold water tub maintained at 15 ± 2 °C (Figure 3a). However, the peripheral blood flow of the finger skin was considerably varied: 37.2 ± 23.2, 43.0 ± 26.0, and 32.8 ± 23.8 mL/min after ingestion of either a high dose or a low dose of the HEPT7G/βCD inclusion complex or placebo, respectively (see Figure 3b). Upon immersion in cold water, a significant decrease in blood flow and skin temperature was noticed due to cold-stimulated stress. Just after withdrawal of the cold stress (t = 0), the mean finger skin temperature was almost the same for all the treatments (Figure 3c); however, a variation in the finger skin peripheral blood flow (Figure 3d) was clearly observed for both high dose (21.8 ± 17.9 mL/min) and low dose (20.8 ± 14.7 mL/min) of HEPT7G/βCD inclusion complex intakes compared to placebo (14.8 ± 8.7 mL/min).

**Figure 4:**
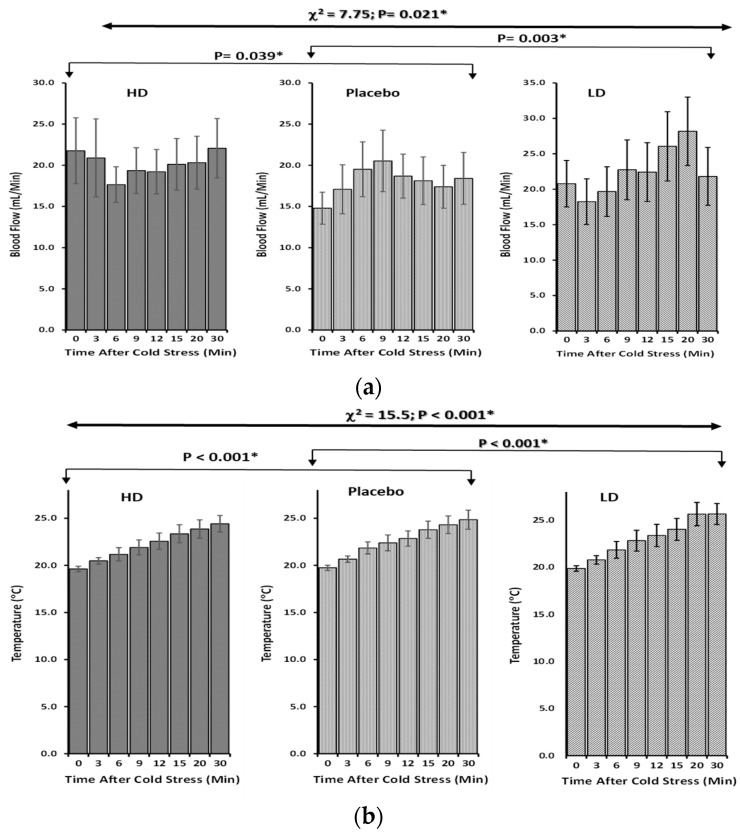

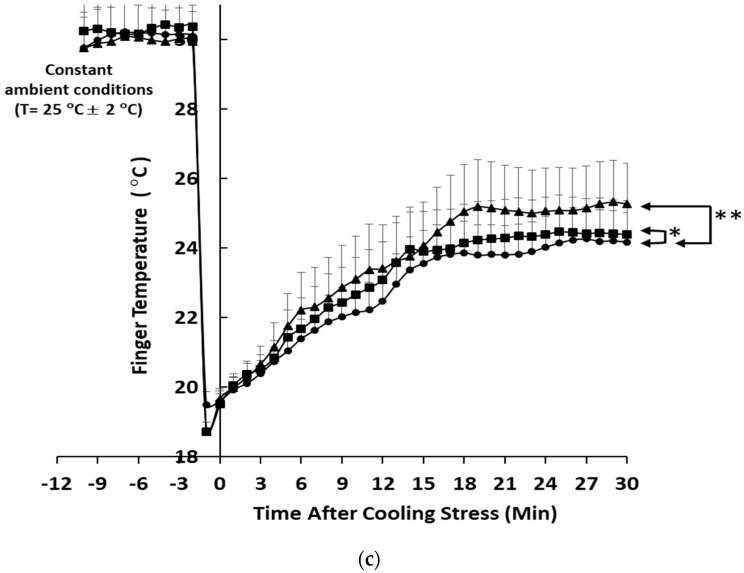
The skin peripheral blood flow and temperature profiles of the study subjects during rewarming (recovery) after cold−stimulate stress in healthy human subjects. The bar plot of mean ± SEM values of profiles is presented for the placebo, high−dose (HD), and low−dose (LD) HEPT7G/βCD inclusion complex intake groups. (**a**) Blood flow recovery; (**b**) temperature recovery; and (**c**) stratified analysis after excluding the effect of hemoconcentration during the localized cooling (*n* = 12; hemoglobin levels > 13.2 g/dL). Significant difference: high dose (*p* < 0.05 *; square) and low dose (*p* < 0.01 **; triangle) of HEPT7G/βCD inclusion complex intakes compared to placebo (circle). Statistics: Non−parametric Friedman rank test with repeated measures, and post−hoc pairwise comparisons using the Durbin−Conover test.

Furthermore, the measured finger skin blood flow, conducted during the described set of experiments, showed an effective increasing pattern during the rewarming after the ingestion of both HEPT7G/βCD doses compared to the placebo. There was a significant increase in blood flow, which led to a relatively effective recovery of skin temperature. With placebo intake, the finger skin blood flow remained lower after a brief period of temporary increase. On the other hand, the variation in the time delay of an increase in blood flow during rewarming was prominent in high-dose intake compared to low-dose. A non-parametric Friedman rank test with repeated measures revealed a significant overall difference in finger skin blood flow among the three treatments [χ^2^ = 7.57, *p* = 0.021 *; *n* = 20]. Furthermore, the post-hoc pairwise comparison using the Durbin–Conover test indicated that both the high-dose (Z = 2.28, *p* = 0.039 *) and low-dose (Z = 3.58, *p* = 0.003 *) intakes had significantly higher scores than placebo consumption (Figure 4a). Although greater efficacy was noticed during low-dose intake than high-dose intake when compared to placebo consumption, no significant pairwise comparison (Z = 1.3, *p* = 0.214) was observed between high-dose and low-dose ingestion (see Table 2).

The finger skin temperature before the intake of either varied doses of HEPT7G/βCD inclusion complex or placebo was almost in the constant range of temperature (31.5 °C to 35.7 °C), reflecting the different individual biological responses to the ambient environment, and was not statistically significant. Upon immersion into cold water (15 ± 2 °C), the dynamic responses of the finger skin temperature were unaltered during the exposure to cold stress, and the finger skin temperature decreased, regardless of treatments or placebo intakes. After the withdrawal from cold stress, during the rewarming (recovery) period, a gradual increase in the finger skin temperature was recorded for placebo as well as for HEPT7G/βCD inclusion complex consumptions. For placebo intake, after a brief period of quick recovery of finger skin temperature, the rate of rewarming was comparatively slower than low-dose consumption of the HEPT7G/βCD inclusion complex. Meanwhile, the rewarming was somewhat reduced with high-dose HEPT7G/βCD inclusion complex ingestion without negatively affecting the finger skin blood flow (Table 3).

The result of the non-parametric Friedman rank test with repeated measures indicated a significant overall difference in rewarming efficacy in finger skin temperature among the three treatments (χ^2^ = 15.5, *p* < 0.001 *; *n* = 20). Again, the post-hoc pairwise comparison using the Durbin–Conover test revealed that low-dose (Z = 9.9, *p* < 0.001 *) HEPT7G/βCD inclusion complex consumption had significantly higher scores when compared to placebo intake (Figure 4b). In addition, greater efficacy and a significant pairwise comparison (Z = 21.92, *p* < 0.001 *) were observed between high-dose and low-dose ingestion (see Table 3). However, in a stratified analysis (*n* = 12), after excluding the effect of hemoconcentration during the localized cooling, wherein eight female subjects were excluded from the analysis due to their below-normal range hemoglobin levels (<13.2 g/dL), a significant difference in the variation in peripheral skin temperature of the finger was noticed for both high dose (*p* < 0.05 *) and low dose (*p* < 0.01 **) of HEPT7G/βCD inclusion complex intakes compared to placebo (see Figure 4c).

**Figure 5:**
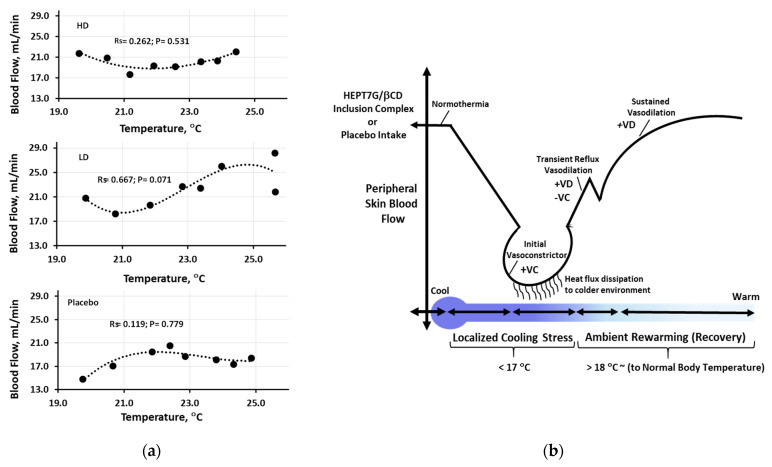
(**a**) The Spearman correlation analysis to examine the relationship between peripheral skin temperature and blood flow for placebo, high−dose (HD), and low−dose (LD) HEPT7G/βCD inclusion complex intake groups (*Significant, p ≤ 0.05; nearly significant* (*trending*), *p ≤ 0.10*). (**b**) The fall in skin temperature causes a reflexive rise in sympathetic active vasoconstrictor nerve activity, which lowers blood flow and conserves body heat. An increase in skin temperature during the rewarming process induces a reflexive increase in sympathetic active vasodilator nerve activity to enhance peripheral skin blood flow.

The Spearman correlation analysis was conducted to examine the relationship between skin temperature and peripheral blood flow (Figure 5a). The results indicated a non-significant correlation between the means of the two above-mentioned variables upon consumption of the varied doses of HEPT7G/βCD inclusion complex (*high dose*: R_s_ = 0.0.262, *p* = 0.531; *low dose*: R_s_ =0.667, *p* = 0.071) and the placebo (R_s_ = 0.119, *p* = 0.779), suggesting a relatively strong positive association between skin temperature and peripheral blood flow for HEPT7G/βCD inclusion complex treatments compared to placebo. This finding may have important implications for understanding the underlying mechanisms that drive the skin temperature and skin peripheral blood flow variables and their relationship, and it may provide support for the hypothesis that the HEPT7G/βCD inclusion complex has a positive impact on peripheral blood flow (Figure 5a).

Further, no sequence effects of the respective high and low doses of HEPT7G/βCD inclusion complex and placebo consumption on peripheral skin blood flow (mL/min) and skin temperature (°C) were noticed between the geometric mean of the three groups after the randomization during the study period. A non-parametric Friedman rank test with repeated measures revealed a significant overall difference for all three groups in both skin blood flow (*Group A*: χ^2^ = 16.0, *p* = 0.002 *, *n* = 7; *Group B*: χ^2^ = 14.3, *p* < 0.001 *, *n* = 7; *Group C*: χ^2^ = 7.0, *p* = 0.030 *; *n* = 6) and skin temperature (*Group A*: χ^2^ = 16.0, *p* = 0.001 **, *n* = 7; *Group B*: χ^2^ = 9.75, *p* < 0.008 *, *n* = 7; *Group C*: χ^2^ = 6.85, *p* = 0.033 *; *n* = 6) (see Tables S2 and S3, respectively). In addition, no period effects on the peripheral blood flow (mL/min) and skin temperature (°C) were noticed for the geometric mean of three doses (high and low doses of HEPT7G/βCD inclusion complex and placebo) after the randomization of subjects in groups (Group A, Group B, and Group C) during the study periods (i.e., *Period 1*, *Period 2*, and *Period 3*). A non-parametric Friedman rank test with repeated measures showed no significant overall statistical difference for all three doses consumed by respective groups in both skin blood flow (*High dose*: χ^2^ = 3.25, *p* = 0.197, *n* = 7; *Low dose*: χ^2^ = 6.31, *p* = 0.064, *n* = 7; *Placebo*: χ^2^ = 4.01, *p* = 0.135, *n* = 6) and skin temperature (*High dose*: χ^2^ = 3.26, *p* = 0.196, *n* = 7; *Low dose*: χ^2^ = 5.73, *p* = 0.057, *n* = 7; *Placebo*: χ^2^ = 1.10, *p* = 0.294, *n* = 6) (see Tables S4 and S5, respectively).

### 3.3. Cold Sensation Response: A Subjective Questionnaire

The results of the subjective questionnaire during the cold-stimulated stress experiments estimate the responses to the degree of coldness experienced by the study subjects using a visual analog scale (VAS). The values of the cold sensation response to the fingertip, hand, and whole body are presented in Table 4.

A non-parametric Friedman rank test with repeated measures revealed a nonsignificant overall difference in cold sensation response for the fingertip (χ^2^ = 7.57, *p* = 0.057; *n* = 20) and hand (χ^2^ = 0.429, *p* = 0.807; *n* = 20) among the three treatments. In addition, post-hoc pairwise comparisons using the Durbin–Conover test indicate that both high-dose (fingertip: Z = 1.49, *p* = 0.088; hand: Z = 0.01, *p* = 1.0) and low-dose (fingertip: Z = 2.38, *p* = 0.055; hand: Z = 0.51, *p* = 0.505) intakes also had non-significant scores compared to placebo consumption. While the cold sensation response to the whole body showed a significant overall difference (χ^2^ = 6.50, *p* = 0.039 *; *n* = 20) among the three treatments. Further, post-hoc pairwise comparisons using the Durbin–Conover test indicate that both the high-dose (Z = 3.54, *p* = 0.012 *) and low-dose (Z = 4.95, *p* = 0.003 *) intakes revealed a significant difference in the scores when compared to placebo (see Table 4).

## 4. Discussion

The main objective of the present study was to gain more physiological and mechanistic insights into the underlying processes of localized cold-stimulate stress upon varied doses of highly bioavailable HEPT7G/βCD inclusion complex consumption. The major metabolites of hesperetin in human plasma are conjugated hesperetin-glucuronides that can be hydrolyzed at the vascular level by β-glucuronidase (i.e., deconjugated) in several tissues, releasing the parent hesperetin aglycone, which accumulates intracellularly and enters blood circulation [39,40]. Wherein, the plasma kinetics of hesperetin-glucuronides mean plasma concentration-time profile (AUC) of total hesperetin metabolites of the HEPT7G/βCD inclusion complex showed over a 100-fold higher AUC than placebo [35]. The solubility of HEPT7G may be useful to improve the health benefits of this proprietary HEPT7G/βCD inclusion complex formulation [34]. The findings indicate that appropriate intervention of the HEPT7G/βCD inclusion complex could have a beneficial effect on blood flow through the modulation of local cooling stimulus on vasodilation and vasoconstriction in healthy individuals. The cold-stimulate vasoconstriction is initiated by the mitochondrial generation of reactive oxygen species (ROS), which refers to the narrowing of blood vessels, which decreases blood flow. Upon immersion of the hand into cold water, the decreasing local skin temperature causes temperature-dependent cutaneous vasoconstriction, resulting in decreased cutaneous skin blood flow [41,42,43]. During vasoconstriction, the high surface area-to-volume ratio and the skin temperature tend to exponentially decrease to the targeted lower temperature level to induce a cold sensation. Such initial vasoconstriction is usually stimulated not based on the skin temperature threshold but rather on a certain threshold in the rate of heat dissipation from the skin to the surrounding environment. This may suggest that the decrease in skin blood flow rate might start earlier than the decrease in skin temperature. Even at comparable skin temperatures, additional factors such as skin hydration and free radical exposure can change blood flow. Therefore, in terms of mechanism of action, cutaneous blood flow could be regulated by two branches of the sympathetic nervous system: a noradrenergic vasoconstrictor system and a cholinergic active vasodilator system. The fall in skin temperature causes a reflexive rise in sympathetic active vasoconstrictor nerve activity, which lowers blood flow and conserves body heat. Whereas the increase in skin temperature during the rewarming process induces a reflexive increase in sympathetic active vasodilator nerve activity to enhance blood flow to the skin (see Figure 5b).

The HEPT7G/βCD inclusion complex administration may inhibit the non-adrenergic vasoconstrictor response to cold-stimulate stress in the human skin because non-adrenergic involvement is predominant in sudden quenching [44,45,46]. Additionally, gender differences may be influential, especially if the cold sensitivity is of short duration [47]. On the other hand, vasodilation refers to the widening of blood vessels, which increases blood flow. It should be stated here that the local cold-stimulate stimulus vasodilation in the finger skin is possibly dependent on the heat dissipation rate or the rate of rewarming, and may cause significant non-adrenergic vasodilatation in the early phase of rewarming [48,49], using the latent heat that represents the amount of dissipation heat required to trigger the rewarming process. After withdrawing the cold-stimulate stress, the vasoconstriction may be interrupted, resulting in periods of vasodilation, which correspond to an increase in peripheral skin temperature, possibly by activation of cold-sensitive neurons that eventually release the norepinephrine from sympathetic cutaneous vasoconstrictor nerves [50,51]. Intensively, the HEPT7G/βCD inclusion complex was effective in keeping the peripheral skin blood flow in accordance with inhibition of the decrease in finger skin temperature as well as in recovering the skin temperature. Additionally, the localized cooling effects on the hematologic system consist of a simultaneous increase in the subjects’ hemoglobin and hematocrit levels due to hemoconcentration defined via an increase in the proportion of red blood cells relative to the plasma [52]. Therefore, among the subjects (hemoglobin level >13.2 g/dL) with the annulled effect of hemoconcentration, a stratified analysis of the variation in peripheral skin temperature of the finger also confirmed a dose-dependently significant effect of the HEPT7G/βCD inclusion complex compared to placebo.

Moreover, hesperetin monoglucosides and aglycone hesperetin have also been shown to have antioxidant and anti-inflammatory properties [53,54,55,56,57,58,59] that may help reduce inflammation and oxidative stress in the body, which can lead to cold sensations. Therefore, we have hypothesized that increased quenching of ROS by HEPT7G/βCD inclusion complex intake may act to decrease the efficacy of the non-adrenergic receptors, thus decreasing the cutaneous vasoconstrictor response during cold-stimulate stress. The exact mechanism by which hesperetin can help reduce cold sensations in the body is not yet fully understood. However, there are several theories and mechanisms that may explain how hesperetin may work to improve blood circulation and reduce feelings of coldness. In the case of non-adrenergic involvement, functional NOS could play an important role during cold-stimulated stress in the human skin [45,48,60]. One possible mechanism is that hesperetin may help promote the production of NO, a compound that relaxes blood vessels and improves blood flow. Generally, NO is a vasodilator that relaxes the smooth muscle cells in the blood vessel walls, leading to an increase in blood flow [28,61,62]. This improved skin blood flow can help warm up the body and reduce feelings of coldness. Secondly, hesperetin metabolites, mostly present as conjugated metabolites, have been found to be beneficial for the vascular system, particularly to improve endothelial function. Takumi et al. (2012) presented that plasma hesperetin metabolites can increase NO release from endothelial cells by inhibiting NADPH oxidase activity, which is also suggested to be a pivotal vasoprotective molecule [33,63]. Lui et al. reported that hesperetin up-regulated endothelium nitric oxide synthase (eNOS) expression and enhanced NO release from endothelial cells [29]. The endothelium is the inner lining of the blood vessels and plays a key role in regulating blood flow through an arterial vasomotor response mediated by the release of vasoconstriction and vasodilatory chemical substances from the endothelium. Hesperetin metabolites help to improve vascular endothelial function by reducing oxidative stress and inflammation, improving the production of endothelial-derived relaxing factors, and therefore reducing cardiovascular risk [22,28,30,56,64,65]. In addition, hesperetin has been shown to help regulate arterial blood pressure and cardiovascular functions by inhibiting the activity of the angiotensin-converting enzyme (ACE), which is responsible for electrolyte balance and raising blood pressure. By reducing ACE activity, hesperetin metabolites can help lower blood pressure, improving skin blood flow in the system [53,66,67]. The results of the present study also demonstrate that the HEPT7G/CD inclusion complex administration could promote blood flow and keep the peripheral temperature higher.

Furthermore, transient receptor potential (TRP) channels are known as highly sensitive molecular thermometers and have been identified to have a role in regulating skin blood flow. They are expressed in vascular smooth muscle cells and endothelial cells, which are important in controlling blood vessel diameter [68]. TRP channels are a family of ion channels found on cell membranes that are involved in the sensation of temperature changes in the sympathetic nervous system and other environmental stimuli [69,70,71]. Cutaneous primary sensory neurons involved in thermosensation include both non-myelinated C fibers and thinly myelinated Aδ-fibers linked to peripheral skin tissues [72], which upon depolarization by TRP channels, convey electrical signals from the skin periphery to the CNS, eliciting temperature sensing in human subjects. It is widely accepted that some TRP channels (TRPM8, TRPA1, and TRPC5) are specifically involved in sensing cold temperatures [73,74]. Particularly, the TRP melastatin-8 (TRPM8) channel is activated by temperatures between 25 °C and 16 °C and is responsible for the sensation of coolness or coldness [75,76]. It is also found in sensory nerves in the skin, and its activation triggers a chain of signals and relays the information to the brain, which leads to the perception of cold [77]. In addition to TRPM8, a nonselective cationic transient receptor potential Ankyrin 1 (TRPA1) channel that is activated by cold below 17 °C in some expression systems [78,79] but alone does not render neurons cold-sensitive, could have also been indirectly activated [80].

**Figure 6:**
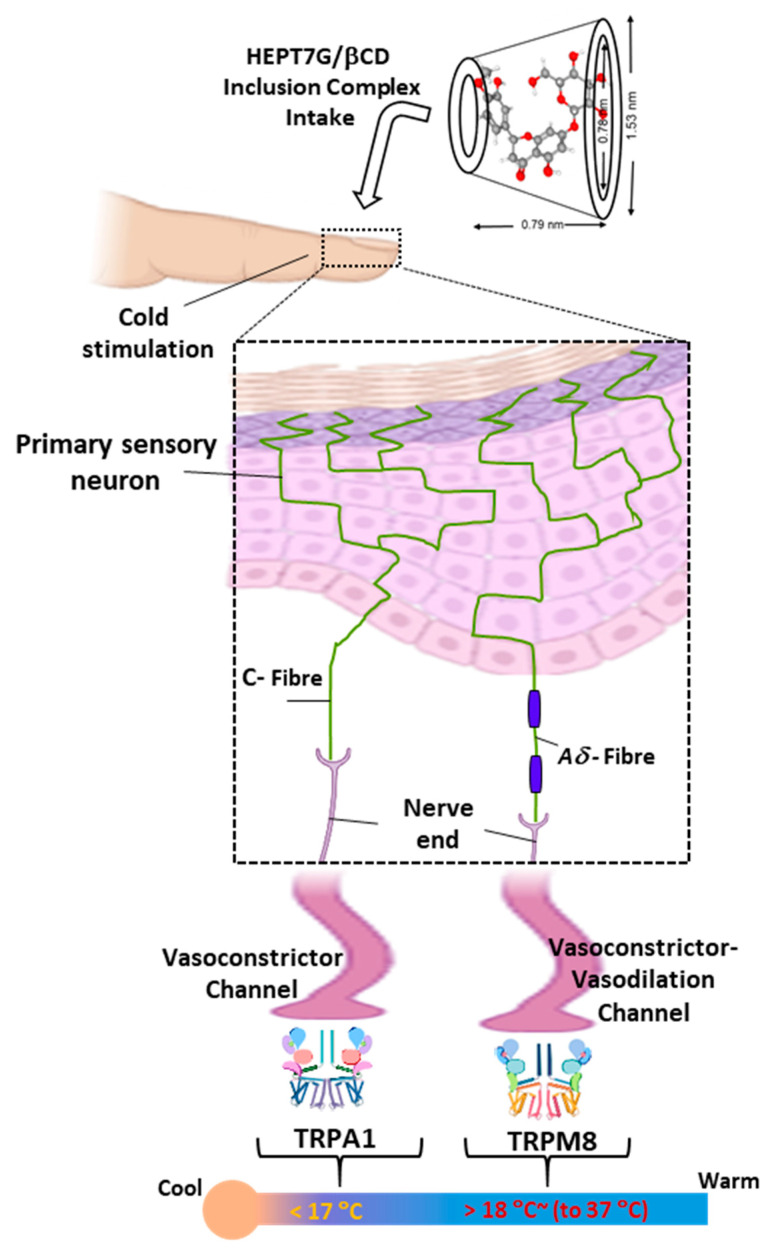
An overview of thermosensation and molecular response mechanisms. The cutaneous primary somatosensory neurons (C-fibers and Aδ-fibers) enact the cold sensation responses at thermal thresholds and thermos-neutral skin temperatures. HEPT7G/βCD inclusion complex intake modulates the balance between vasoconstrictor and vasodilator for improved peripheral blood flow.

Upon immersion of the hand into a cold water tank maintained at 15 ± 2 °C, the TRP channels could sense changes in temperature (see Figure 6), which can trigger blood vessel constriction (vasoconstriction, i.e., narrowing of blood vessels) or control blood vessel dilation (vasodilation or widening of blood vessels). Hesperetin metabolites could have improved the cold sensation by simultaneously activating TRPM8 and TRPA1 transient receptor potential channels [81,82,83]. Wherein, when the TRPM8 channel is activated, it can lead to vasodilation, or the widening of blood vessels, which can increase or improve skin blood flow and reduce blood pressure. Moreover, despite no complaints of decreased metabolism of whole-body cold sensation after the localized cold-stimulate stress challenge at baseline, the participants reported improved skin blood flow recovery based on their VAS scores of cold tolerance after consumption of varied doses of HEPT7G/βCD inclusion complex.

## 5. Conclusions

Cold exposure is directly related to the peripheral skin blood flow behavior, wherein vasoconstriction reduces the cutaneous blood flow to protect the skin against heat loss, tissue damage by the cold, and thus maintains a stable body temperature. It is further followed by vasodilation, which will prevent hypoxic damage. Thus, blood flow is a balance between multiple factors that cause vasodilation and vasoconstriction. To conclude, we have studied a localized cold-stimulated response and spontaneous rewarming to detect the cold sensation among healthy humans after HEPT7G/βCD inclusion complex consumption. The present findings demonstrate that the skin temperature and skin blood flow responses during localized cooling and spontaneous rewarming were also transferable to whole-body conditioning and ameliorated the cold sensation responses. This provides grounds for believing that hesperetin aglycone derived from deconjugation of hesperetin glucuronide metabolites after acute ingestion of rather small doses of highly bioactive HEPT7G/βCD inclusion complex (150 mg and 300 mg SunActive^®^ HES/HCD contains 19.5 mg and 39 mg HEPT7G, respectively) exerted a vasodilator effect in humans in a dose-dependent manner. Such a functional change could be associated with an increase in active hesperetin metabolites responsible for the variable dependence of hesperetin on endothelial NO responses. However, answering every question is crucial for completing our understanding of the mechanisms that underlie cold sensation improvement by hesperetin metabolites after varied doses of consumption of the HEPT7G/βCD inclusion complex. Further evidence and research may provide much-needed responses to comprehend the use of flavonoid-containing nutraceutical interventions in humans that either suffer from hypersensitivity to the cold or its comorbid symptoms.

## Figures and Tables

**Table 1:** Characteristic features and blood biomarker details of the study subjects at baseline.

Characteristics Parameters	Group A	Group B	Group C	All Subjects
	(*n* = 7)	(*n* = 7)	(*n* = 6)	(*n* = 20)
Gender (M/F)	2/5	1/6	1/5	4/16
Age (Years)	49.00 ± 7.14	46.00 ± 10.75	47.83 ± 8.13	47.60 ± 8.47
Height (Cm)	163.43 ± 6.83	160.29 ± 5.57	157.95 ± 5.23	160.77 ± 6.08
Weight (Kg)	57.39 ± 7.81	58.17 ± 6.13	55.02 ± 6.04	56.95 ± 6.52
BMI (Kg/m^2^)	21.43 ± 1.79	22.70 ± 2.79	22.10 ± 2.72	22.07 ± 2.39
Systolic blood pressure (mmHg)	119.86 ± 6.07	118.14 ± 6.79	116.67 ± 9.18	118.30 ± 7.08
Diastolic blood pressure (mmHg)	78.71 ± 6.02	74.29 ± 4.39	77.50 ± 6.28	76.80 ± 5.63
Pulse rate (bpm)	63.00 ± 6.56	63.86 ± 12.35	75.83 ± 7.03	67.15 ± 10.43
Blood flow recovery ratio	0.30 ± 0.69	0.40 ± 1.34	0.28 ± 0.64	0.33 ± 0.91
Skin temperature recovery ratio	0.29 ± 0.16	0.26 ± 0.10	0.24 ± 0.06	0.26 ± 0.11
Total protein (g/dL)	7.17 ± 0.39	7.06 ± 0.42	7.22 ± 0.33	7.15 ± 0.37
Total bilirubin (mg/dL)	0.81 ± 0.30	0.76 ± 0.14	0.82 ± 0.30	0.80 ± 0.24
ALP (U/L)	67.71 ± 23.65	56.00 ± 12.45	82.50 ± 15.22	68.05 ± 20.15
LD (U/L)	151.8 ± 18.78	156.4 ± 26.15	164.7 ± 13.59	157.3 ± 20.11
AST (GOT) (U/L)	20.14 ± 3.34	18.29 ± 3.82	19.50 ± 4.23	19.30 ± 3.67
ALT (GPT) (U/L)	13.71 ± 5.09	16.14 ± 5.87	17.67 ± 6.65	15.75 ± 5.78
γ-GT (γ-GTP) (U/L)	18.29 ± 8.86	20.29 ± 7.80	41.00 ± 24.92	25.80 ± 17.68
Creatine kinase (U/L)	84.71 ± 25.71	86.29 ± 41.95	73.00 ± 29.82	81.75 ± 32.15
Total cholesterol (mg/dL)	210.1± 19.18	189.7 ± 42.59	208.5 ± 26.49	202.5 ± 31.09
Triglycerides (mg/dL)	68.14 ± 17.39	83.29 ± 86.70	56.50 ± 31.80	69.95 ± 53.47
HDL cholesterol (mg/dL)	70.43 ± 12.49	70.43 ± 19.49	73.67 ± 15.13	71.40 ± 15.23
LDL cholesterol (mg/dL)	128.7 ± 18.63	101.1 ± 36.70	123.2 ± 17.05	117.4 ± 27.69
Urea nitrogen (mg/dL)	11.57 ± 2.32	14.01 ± 5.34	12.93 ± 3.38	12.84 ± 3.85
Creatinine (mg/dL)	0.67 ± 0.13	0.61 ± 0.15	0.59 ± 0.12	0.63 ± 0.13
Uric acid (mg/dL)	4.73 ± 1.55	4.24 ± 1.05	4.37 ± 0.72	4.45 ± 1.14
Glucose (g/dL)	81.71 ± 5.74	79.57 ± 5.62	81.50 ± 7.40	80.9 ± 5.98
HbA1C (%)	5.30 ± 0.26	5.39 ± 0.21	5.28 ± 0.38	5.33 ± 0.28
Leukocytes (WBC) (µL^−1^)	5666 ± 1312	5710 ± 1466	6038 ± 1663	5793 ± 1406
Erythrocytes (RBC) × 10^4^ (µL^−1^)	465.0 ± 32.11	446.6 ± 27.20	464.7 ± 24.39	458.5 ± 28.21
Hemoglobin (dL)	14.10 ± 1.26	13.21 ± 0.38	13.95 ± 0.56	13.75 ± 0.89
Hct (%)	43.66 ± 3.60	40.04 ± 1.87	42.53 ± 1.81	42.06 ± 2.92
MCV (fL)	94.0 ± 5.48	90.00 ± 6.66	91.83 ± 5.15	91.95 ± 5.78
MCH (pg)	30.3 ± 0.1.26	29.70 ± 1.94	30.07 ± 1.87	30.02 ± 1.64
MCHC (%)	32.31 ± 1.10	33.03 ± 0.62	32.83 ± 0.81	32.72 ± 0.88
Platelet 10^4^ (µL^−1^)	28.67 ± 11.09	24.69 ± 2.61	28.92 ± 10.28	27.35 ± 8.53

Significance *p* ≤ 0.05 (no significant differences were observed among groups).

**Table 2:** A non-parametric Friedman rank test with repeated measures followed by post-hoc pairwise comparison using the Durbin–Conover test revealed a significant overall difference in peripheral skin finger blood flow (mL/min) during the temperature recovery among the HEPT7G/βCD inclusion complex treatments compared to placebo.

Time after Cooling Stress	HEPT7G/βCD Inclusion Complex		Placebo (β-CD)
	High Dose (*n* = 20)	Low Dose (*n* = 20)	(*n* = 20)
*Min*	*Mean ± SD*	*Mean ± SD*	*Mean ± SD*
0	21.77 ± 17.89	20.79 ± 14.66	14.80 ± 8.67
3	20.90 ± 21.21	18.25 ± 14.45	17.08 ± 13.31
6	17.66 ± 9.71	19.67 ± 15.60	19.52 ± 14.89
9	19.35 ± 12.45	22.75 ± 18.87	20.53 ± 16.72
12	19.21 ± 12.08	22.43 ± 18.54	18.69 ± 11.96
15	20.12 ± 13.99	26.06 ± 21.90	18.13 ± 12.95
20	20.32 ± 14.33	28.17 ± 21.61	17.39 ± 11.61
30	22.07 ± 16.11	21.81 ± 18.27	18.41 ± 14.10
***Friedman Rank Test*** ***Repeated Measure* (*Non-Parametric*)**	χ^2^-Value: 7.75 *p*-Value: 0.021 *		
***Durbin–Conover (Post-hoc Test) †***	*HD* vs. *P*	*LD* vs. *P*	*HD* vs. *LD*
Z-*Score*	*2.28*	*3.58*	*1.3*
*p*-*Value*	**0.039** *	**0.003** *	*0.214*

† Pairwise comparison (between groups); * significance *p* ≤ 0.05; HD = High Dose; LD = Low Dose; *P* = Placebo.

**Table 3:** A non-parametric Friedman rank test with repeated measures followed by post-hoc pairwise comparison using the Durbin–Conover test revealed a significant overall difference in peripheral skin finger temperature (°C) during ambient rewarming (recovery) among the HEPT7G/βCD inclusion complex treatments compared to placebo.

Time after Cooling Stress	HEPT7G/βCD Inclusion Complex		Placebo (β-CD)
	High Dose (*n* = 20)	Low Dose (*n* = 20)	(*n* = 20)
*Min*	*Mean ± SD*	*Mean ± SD*	*Mean ± SD*
0	19.62 ± 1.30	19.87 ± 1.43	19.80 ± 1.25
3	20.48 ± 1.53	20.79 ± 2.03	20.73 ± 1.53
6	21.18 ± 3.15	21.85 ± 3.99	21.96 ± 2.91
9	21.91 ± 3.56	22.83 ±4.94	22.53 ± 3.75
12	22.57 ± 3.92	23.38 ± 5.30	22.99 ± 3.62
15	23.35 ± 4.29	24.03 ± 5.19	23.96 ± 4.07
20	23.86 ± 4.34	25.65 ± 5.53	24.52 ± 4.17
30	24.42 ± 3.92	25.67 ± 4.99	25.09 ± 4.50
***Friedman Rank Test*** ***Repeated Measure*** **(*Non-Parametric*)**	χ^2^-Value: 15.5 *p*-Value: <0.001 **		
***Durbin-Conover*** **(*Post-hoc Test*) †**	*HD* vs. *P*	*LD* vs. *P*	*HD* vs. *LD*
Z-*Score*	*12.02*	*9.9*	*21.92*
*p*-*Value*	**<0.001** **	**<0.001** **	**<0.001** **

† Pairwise comparison (between groups); ** highly significant *p* ≤ 0.001; HD = High Dose; LD = Low Dose; *P* = Placebo.

**Table 4:** Subjective questionnaire scores (*geometric mean ± SD*) during the cold-stimulate stress experiments estimate the responses to the degree of coldness experienced to the fingertip, hand, and whole body by the study subjects using a visual analog scale (VAS). A non−parametric Friedman rank test with repeated measures followed by post-hoc pairwise comparison using Durbin–Conover presented overall differences during ambient rewarming (temperature recovery) among the HEPT7G/βCD inclusion complex treatments compared to placebo.

Questionnaire Description (*Degree of Coldness*)	Time after Cooling Stress	HEPT7G/βCD Inclusion Complex		Placebo (β−CD)	Friedman Rank Test Repeated Measure (Non−Parametric)	† Durbin–Conover (Post−Hoc Test)		
		High Dose (*n* = 20)	Low Dose (*n* = 20)	(*n* = 20)		*Z-Score*; *p-Value*		
	*Min*	*Geo*−*Mean ± SD*	*Geo*−*Mean ± SD*	*Geo*−*Mean ± SD*		*HD* vs. *P*	*LD* vs. *P*	*HD* vs. *LD*
	0	61.69 ± 28.43	62.83 ± 30.24	64.38 ± 26.86				
*Fingertip*	15	62.17 ± 24.81	56.62 ± 28.20	54.92 ± 27.56	χ^2^-Value = 5.73	1.49	2.38	3.86
	20	54.31 ± 26.15	51.58 ± 27.15	51.67 ± 32.28	*p*-Value = **0.057 ^#^**	**0.088 ^#^**	**0.055 ^#^**	0.008 *
	30	52.33 ± 29.85	48.38 ± 30.34	47.70 ± 33.78				
	0	63.85 ± 26.21	61.52 ± 31.31	60.99 ± 28.21				
*Hand*	15	54.95 ± 29.28	53.99 ± 28.47	41.01 ± 32.37	χ^2^-Value = 0.429	0.00	0.535	0.505
	20	40.23 ± 33.34	43.90 ± 29.68	44.97 ± 33.47	*p*-Value = *0.807*	1.00	0.505	0.632
	30	42.52 ± 31.99	38.18 ± 32.03	39.54 ± 32.63				
	0	36.39 ± 25.50	26.77 ± 34.68	44.57 ± 28.93				
*Body*	15	36.88 ± 31.02	29.31 ± 32.03	40.07 ± 31.01	χ^2^-Value = 6.50	3.54	4.95	1.41
	20	33.20 ± 32.19	27.30 ± 31.78	36.26 ± 29.69	*p*-Value = 0.039 *	0.012 *	0.003 *	0.207
	30	35.70 ± 32.02	25.00 ± 31.74	33.43 ± 31.07				

† Pairwise comparison (between groups); * significance *p* ≤ 0.05; HD = High Dose; LD = Low Dose; *P* = Placebo; ^#^ Nearly Significant.

## Data Availability

All data generated in this study are included in this manuscript. Data will be made available on request.

## Supplementary Materials

The following supporting information can be downloaded at: https://www.mdpi.com/article/10.3390/nu15173702/s1, Figure S1: The chemical structure of hesperidin, hesperetin-7-*O*-glucoside, and hesperetin (aglycone). Table S1: Terasawa’s Cold Sensitivity (hiesho) Diagnostic Criteria; Table S2: No sequence effects of the respective high and low doses of HEPT7G/βCD inclusion complex and placebo consumption on the peripheral skin blood flow (i.e., primary outcome, mL/min) were noticed between the geometric mean of the three groups after the randomization during the study period. A non-parametric Friedman rank test with repeated measures revealed a significant overall difference for all three groups; Table S3: No sequence effects of the respective high and low doses of HEPT7G/βCD inclusion complex and placebo consumption on the peripheral skin temperature (°C) were noticed between the geometric mean of the three groups after the randomization during the study period. A non-parametric Friedman rank test with repeated measures revealed a significant overall difference for all three groups; Table S4: No period effects on the peripheral skin blood flow (mL/min) were noticed for the geometric mean of three doses (high and low doses of HEPT7G/βCD inclusion complex and placebo) after the randomization of subjects in groups (Group A, Group B, and Group C) during the study periods. A non-parametric Friedman rank test with repeated measures showed no significant overall statistical difference for all three doses consumed by the respective groups; Table S5: No period effects on the peripheral skin temperature (°C) were noticed for the geometric mean of three doses (high and low doses of HEPT7G/βCD inclusion complex and placebo) after the randomization of subjects in groups (Group A, Group B, and Group C) during the study periods. A non-parametric Friedman rank test with repeated measures showed no significant overall statistical difference for all three doses consumed by the respective groups.
