# Supplementary material for: Hesperetin-7-O-glucoside/β-cyclodextrin Inclusion Complex Induces Acute Vasodilator Effect to Inhibit the Cold Sensation Response during Localized Cold-Stimulate Stress in Healthy Human Subjects: A Randomized, Double-Blind, Crossover, and Placebo-Controlled Study
Source: Nutrients. 2023 Aug 24;15(17):3702. doi: 10.3390/nu15173702 (PMC10489958; doi:10.3390/nu15173702)
Supplement: Supplementary file 1 [file nutrients-15-03702-s001.zip › nutrients-2576659-supplementary.pdf]

Supplementary Information

Mahendra P. Kapoor et al. (Nutrients, a MDPI publication)

Total Figure: 1 *(Figure S1)*

Total Tables: 5 *(Tables S1 to S5)*

Figure S1: The chemical structure of hesperidin, hesperetin-7-*O*-glucoside, and hesperetin (aglycone).

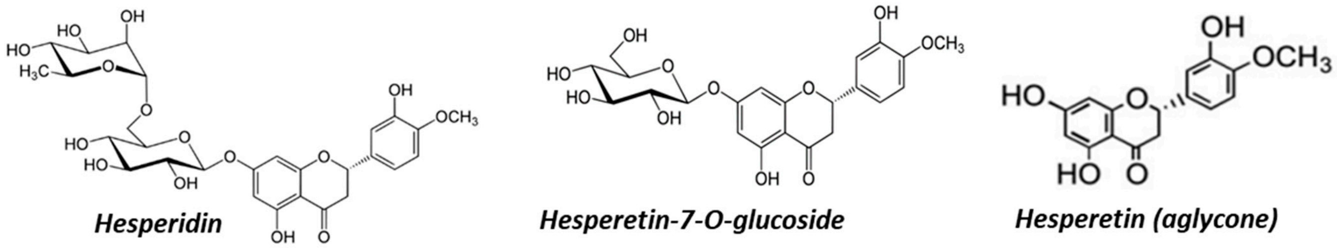

Table S1. Terasawa’s Cold Sensitivity (hiesho) Diagnostic Criteria

Group A (Important questions)

(i) Sensitive to a reduction in environmental temperature than others?

(ii) Feel cold and pain in waist, foot, hand or some parts of the body?

(iii) Need and electric blanket or pocket warmer in winter?

Group B (Reference questions)

(iv) Feel colder in a cold environment than others do?

(v) Wear thicker socks in summer due to coldness?

(vi) Feel cold in an air-conditioned room in summer where most people feel comfortable?

(vii) Need thicker clothes than others do?

(viii) Feel the temperature or the hand and the feet are lower than other parts of the body?

Terasawa’s diagnostic criteria were used to determine whether subjects were suffering from cold sensitivity (hiesho). (1) Subjects were diagnosed as suffering from cold sensitivity if they answered "yes" to more than two important questions in Group A, or (2) to more than one important question in Group A and two reference questions in Group B, or (3) to four or five reference questions in Group B.

Table S2. No sequence effects of the respective high and low doses of HEPT7G/βCD inclusion complex and placebo consumption on the peripheral skin blood flow (*i.e.,* primary outcome, mL/min) were noticed between the geometric mean of the three groups after the randomization during the study period. A non-parametric Friedman rank test with repeated measures revealed a significant overall difference for all three groups.

**Time After Cooling Stress**	**Group A (N = 7)**			**Group B (N = 7)**			**Group C (N = 6)**		
	**HD**-*P1*	**LD**-*P2*	**P**-*P3*	**HD**-*P3*	**LD**-*P1*	**P**-*P2*	**HD**-*P2*	**LD**-*P3*	**P**-*P1*
Minutes	*G-Mean ± SD*	*G-Mean ± SD*	*G-Mean ± SD*	*G-Mean ± SD*	*G-Mean ± SD*	*G-Mean ± SD*	*G-Mean ± SD*	*G-Mean ± SD*	*G-Mean ± SD*
0	11.59 ± 11.64	22.60 ± 16.67	12.09 ± 10.32	15.67 ± 8.16	11.64 ± 10.45	13.63 ± 8.23	28.16 ± 27.18	17.11 ± 16.03	9.87 ± 9.50
3	9.38 ± 11.37	20.32 ± 17.99	10.99 ± 11.16	13.99 ± 5.59	7.83 ± 5.02	13.07 ± 8.41	26.48 ± 32.56	15.56 ± 12.63	17.75 ± 19.22
6	10.72 ± 11.79	20.06 ± 19.31	16.47 ± 14.34	14.84 ± 7.35	9.13 ± 7.61	12.21 ± 12.71	20.60 ± 10.60	16.93 ± 15.26	17.45 ± 18.41
9	11.87 ± 15.31	27.47 ± 22.51	17.46 ± 13.72	16.35 ± 9.03	8.83 ± 9.00	12.86 ± 18.55	20.04 ± 13.44	17.27 ± 15.16	16.78 ± 19.09
12	11.72 ± 13.63	23.33 ± 22.91	16.93 ± 11.00	15.70 ± 7.35	9.47 ± 8.37	14.05 ± 15.13	19.40 ± 14.53	19.57 ± 17.01	15.23 ± 10.17
15	10.17 ± 9.35	22.84 ± 24.92	13.53 ± 11.15	16.11 ± 11.57	10.83 ± 10.19	13.89 ± 16.99	24.86 ± 16.30	26.18 ± 23.91	15.75 ± 10.76
20	10.69 ± 13.07	25.70 ± 21.82	12.91 ± 9.39	17.72 ± 16.72	12.57 ± 13.17	12.41 ± 10.93	21.11 ± 11.57	28.94 ± 24.99	17.93 ± 13.88
30	11.12 ± 13.29	17.64 ± 19.31	12.69 ± 8.24	20.09 ± 21.21	10.01 ± 10.35	13.26 ± 16.99	22.19 ± 10.35	22.74 ± 20.51	18.32 ± 15.69
Friedman Rank Test Repeated Measure (Non-Parametric)	χ^2 – Value: 16.0<br>p – Value: **0.002***			χ^2– Value: 14.3<br>p – Value: **< 0.001****			χ^2 – Value: 7.00<br>p – Value**: 0.030***		

* Significant P < 0.05; Highly Significant p < 0.001**; HD= High Dose; LD = Low Dose; P = Placebo; G-Mean = Geometric Mean; P1= Period 1, P2= Period 2, P3= Period 3

Table S3. No sequence effects of the respective high and low doses of HEPT7G/βCD inclusion complex and placebo consumption on the peripheral skin temperature (°C) were noticed between the geometric mean of the three groups after the randomization during the study period. A non-parametric Friedman rank test with repeated measures revealed a significant overall difference for all three groups.

**Time After Cooling Stress**	**Group A (N = 7)**			**Group B (N = 7)**			**Group C (N = 6)**		
	**HD**-*P1*	**LD**-*P2*	**P**-*P3*	**HD**-*P3*	**LD**-*P1*	**P**-*P2*	**HD**-*P2*	**LD**-*P3*	**P**-*P1*
Minutes	*G-Mean ± SD*	*G-Mean ± SD*	*G-Mean ± SD*	*G-Mean ± SD*	*G-Mean ± SD*	*G-Mean ± SD*	*G-Mean ± SD*	*G-Mean ± SD*	*G-Mean ± SD*
0	18.85 ± 1.42	20.25 ± 0.99	20.12 ± 1.43	20.12 ± 0.93	19.62 ± 1.55	19.38 ± 0.99	19.81 ± 1.34	19.59 ± 1.54	19.62 ± 1.36
3	19.59 ± 1.86	21.03 ± 1.51	20.83 ± 1.48	20.89 ± 0.97	19.96 ± 1.59	20.22 ± 1.60	20.89 ± 1.45	21.19 ± 2.92	20.81 ± 1.69
6	20.54 ± 5.14	22.24 ± 4.09	21.16 ± 1.36	21.04 ± 0.90	20.15 ± 1.38	21.62 ± 4.80	21.46 ± 2.17	22.53 ± 5.66	22.40 ± 2.95
9	21.01 ± 5.08	24.11 ± 5.97	21.49 ± 1.44	21.66 ± 1.37	20.33 ± 1.39	21.83 ± 4.31	22.49 ± 3.72	23.01 ± 5.83	23.32 ± 5.05
12	21.40 ± 5.24	24.86 ± 6.80	22.20 ± 1.91	22.24 ± 1.85	20.76 ± 1.42	22.15 ± 4.05	23.46 ± 4.32	23.33 ± 5.70	23.69 ± 4.84
15	21.81 ± 4.80	25.10 ± 6.67	23.02 ± 2.96	22.89 ± 2.94	21.22 ± 1.48	23.07 ± 4.67	24.69 ± 5.12	24.74 ± 5.43	24.58 ± 4.88
20	22.36 ± 4.77	25.06 ± 6.28	23.71 ± 3.56	23.87 ± 3.74	22.66 ± 3.33	22.98 ± 3.82	24.58 ± 4.92	28.36 ± 5.66	25.61 ± 5.29
30	22.94 ± 4.10	24.57 ± 5.52	24.41 ± 4.39	24.83 ± 3.73	23.49 ± 3.46	23.52 ± 3.91	24.84 ± 4.28	28.27 ± 5.18	25.80 ± 5.60
Friedman Rank Test Repeated Measure (Non-Parametric)	χ^2 – Value: 16.0<br>p – Value: **< 0.001****			χ^2 – Value: 9.75<br>p – Value: **0.008***			χ^2 – Value: 6.85<br>p – Value: **0.033***		

* Significant P < 0.05; Highly Significant p < 0.001**; HD= High Dose; LD = Low Dose; P = Placebo; G-Mean = Geometric Mean; P1= Period 1, P2= Period 2, P3= Period 3

Table S4. No period effects on the peripheral skin blood flow (mL/min) were noticed for the geometric mean of three doses (high and low doses of HEPT7G/βCD inclusion complex and placebo) after the randomization of subjects in groups (Group A, Group B, and Group C) during the study periods. A non-parametric Friedman rank test with repeated measures showed no significant overall statistical difference for all three doses consumed by the respective groups.

Time After Cooling Stress	High Dose (HD)			Low Dose (LD)			Placebo (P)		
	Group A-P1	*Group B-P3*	*Group C-P2*	*Group A-P2*	*Group B-P1*	*Group C-P3*	*Group A-P3*	*Group B-P2*	*Group C-P1*
Minutes	*G-Mean ± SD*	*G-Mean ± SD*	*G-Mean ± SD*	*G-Mean ± SD*	*G-Mean ± SD*	*G-Mean ± SD*	*G-Mean ± SD*	*G-Mean ± SD*	*G-Mean ± SD*
0	11.59 ± 11.64	15.67 ± 8.16	28.16 ± 27.18	11.64 ± 10.45	22.60 ± 16.67	17.11 ± 16.03	12.09 ± 10.32	13.63 ± 8.23	9.87 ± 9.50
3	9.38 ± 11.37	13.99 ± 5.59	26.48 ± 32.56	7.83 ± 5.02	20.32 ± 17.99	15.56 ± 12.63	10.99 ± 11.16	13.07 ± 8.41	17.75 ± 19.22
6	10.72 ± 11.79	14.84 ± 7.35	20.60 ± 10.60	9.13 ± 7.61	20.06 ± 19.31	16.93 ± 15.26	16.47 ± 14.34	12.21 ± 12.71	17.45 ± 18.41
9	11.87 ± 15.31	16.35 ± 9.03	20.04 ± 13.44	8.83 ± 9.00	27.47 ± 22.51	17.27 ± 15.16	17.46 ± 13.72	12.86 ± 18.55	16.78 ± 19.09
12	11.72 ± 13.63	15.70 ± 7.35	19.40 ± 14.53	9.47 ± 8.37	23.33 ± 22.91	19.57 ± 17.01	16.93 ± 11.00	14.05 ± 15.13	15.23 ± 10.17
15	10.17 ± 9.35	16.11 ± 11.57	24.86 ± 16.30	10.83 ± 10.19	22.84 ± 24.92	26.18 ± 23.91	13.53 ± 11.15	13.89 ± 16.99	15.75 ± 10.76
20	10.69 ± 13.07	17.72 ± 16.72	21.11 ± 11.57	12.57 ± 13.17	25.70 ± 21.82	28.94 ± 24.99	12.91 ± 9.39	12.41 ± 10.93	17.93 ± 13.88
30	11.12 ± 13.29	20.09 ± 21.21	22.19 ± 10.35	10.01 ± 10.35	17.64 ± 19.31	22.74 ± 20.51	12.69 ± 8.24	13.26 ± 16.99	18.32 ± 15.69
Friedman Rank Test Repeated Measure (Non-Parametric)	χ^2 – Value: 3.25<br>p – Value: 0.197			χ^2 – Value: 6.31<br>p – Value: 0.064			χ^2 – Value: 4.01<br>p – Value: 0.135		
Durbin-Conover (Post-hoc Test)†	*AP1 vs. BP3*	*AP1 vs. CP2*	*BP3 vs. CP2*	*AP2 vs. BP1*	*AP2 vs. CP3*	*BP1 vs. CP3*	*AP3 vs. BP2*	*AP3 vs. CP1*	*BP2 vs. CP1*
Z – *Score*	*1.31*	*1.83*	*0.524*	*2.28*	*0.966*	*2.31*	*1.08*	*2.16*	*1.08*
p – Value:	*0.211*	*0.088*	*0.609*	*0.091*	*0.350*	*0.089*	*0.298*	*0.099*	*0.298*

† Pairwise Comparison (Between Groups); * Significance $p < 0.05$; Group A (N=7); Group B (N=7); Group C (N=6)

Table S5. No period effects on the peripheral skin temperature (°C) were noticed for the geometric mean of three doses (high and low doses of HEPT7G/βCD inclusion complex and placebo) after the randomization of subjects in groups (Group A, Group B, and Group C) during the study periods. A non-parametric Friedman rank test with repeated measures showed no significant overall statistical difference for all three doses consumed by the respective groups.

**Time After Cooling Stress**	**High Dose (HD)**			**Low Dose (LD)**			**Placebo (P)**		
	Group A-P1	*Group B-P3*	*Group C-P2*	*Group A-P2*	*Group B-P1*	*Group C-P3*	*Group A-P3*	*Group B-P2*	*Group C-P1*
Minutes	*G-Mean ± SD*	*G-Mean ± SD*	*G-Mean ± SD*	*G-Mean ± SD*	*G-Mean ± SD*	*G-Mean ± SD*	*G-Mean ± SD*	*G-Mean ± SD*	*G-Mean ± SD*
0	18.85 ± 1.42	20.12 ± 0.93	19.81 ± 1.34	20.25 ± 0.99	19.62 ± 1.55	19.59 ± 1.54	20.12 ± 1.43	19.38 ± 0.99	19.62 ± 1.36
3	19.59 ± 1.86	20.89 ± 0.97	20.89 ± 1.45	21.03 ± 1.51	19.96 ± 1.59	21.19 ± 2.92	20.83 ± 1.48	20.22 ± 1.60	20.81 ± 1.69
6	20.54 ± 5.14	21.04 ± 0.90	21.46 ± 2.17	22.24 ± 4.09	20.15 ± 1.38	22.53 ± 5.66	21.16 ± 1.36	21.62 ± 4.80	22.40 ± 2.95
9	21.01 ± 5.08	21.66 ± 1.37	22.49 ± 3.72	24.11 ± 5.97	20.33 ± 1.39	23.01 ± 5.83	21.49 ± 1.44	21.83 ± 4.31	23.32 ± 5.05
12	21.40 ± 5.24	22.24 ± 1.85	23.46 ± 4.32	24.86 ± 6.80	20.76 ± 1.42	23.33 ± 5.70	22.20 ± 1.91	22.15 ± 4.05	23.69 ± 4.84
15	21.81 ± 4.80	22.89 ± 2.94	24.69 ± 5.12	25.10 ± 6.67	21.22 ± 1.48	24.74 ± 5.43	23.02 ± 2.96	23.07 ± 4.67	24.58 ± 4.88
20	22.36 ± 4.77	23.87 ± 3.74	24.58 ± 4.92	25.06 ± 6.28	22.66 ± 3.33	28.36 ± 5.66	23.71 ± 3.56	22.98 ± 3.82	25.61 ± 5.29
30	22.94 ± 4.10	24.83 ± 3.73	24.84 ± 4.28	24.57 ± 5.52	23.49 ± 3.46	28.27 ± 5.18	24.41 ± 4.39	23.52 ± 3.91	25.80 ± 5.60
Friedman Rank Test Repeated Measure (Non-Parametric)	χ^2 – Value: 3.26<br>p – Value: 0.196			χ^2 – Value: 5.73<br>p – Value: 0.057			χ^2 – Value: 1.10<br>p – Value: 0.294		
Durbin-Conover (Post-hoc Test)†	*AP1 vs. BP3*	*AP1 vs. CP2*	*BP3 vs. CP2*	*AP2 vs. BP1*	*AP2 vs. CP3*	*BP1 vs. CP3*	*AP3 vs. BP2*	*AP3 vs. CP1*	*BP2 vs. CP1*
Z – *Score*	*1.33*	*3.19*	*0.276*	*3.34*	*0.00*	*3.19*	*0.044*	*1.10*	*2.48*
p – Value:	*0.284*	*0.074*	*0.600*	*0.081*	*1.00*	*0.074*	*0.834*	*0.294*	*0.294*

† Pairwise Comparison (Between Groups); * Significance $p < 0.05$; Group A (N=7); Group B (N=7); Group C (N=6)
